# Laboratory reference intervals influence referral patterns for hemoglobin abnormalities in the Ontario virtual care system

**DOI:** 10.1371/journal.pdig.0000580

**Published:** 2024-08-21

**Authors:** Maud Ahmad, Benjamin Chin-Yee, Ian H. Chin-Yee, Ben Hedley, Cyrus C. Hsia

**Affiliations:** 1 Schulich School of Medicine and Dentistry, London, Ontario, Canada; 2 Division of Hematology, Department of Medicine, London Health Sciences Centre, London, Ontario, Canada; 3 Department of Pathology & Laboratory Medicine, Schulich School of Medicine and Dentistry, Western University, London, Ontario, Canada; The University of Hong Kong, HONG KONG

## Abstract

This retrospective cross-sectional study investigates the impact of laboratory-specific hemoglobin reference intervals on electronic consultation (eConsult) referral patterns for suspected anemia and elevated hemoglobin at a tertiary care center in London, Ontario that serves Southwestern Ontario. The study analyzed referrals through the Ontario Telemedicine Network’s eConsult platform for hemoglobin abnormalities, excluding patients under 18 years old, between July 1, 2019, and June 30, 2023.The main outcome measures were influence of hemoglobin reference intervals on the referral patterns for suspected anemia and elevated hemoglobin, as well as the extent of pre-referral laboratory testing. Of the 619 eConsults reviewed, 251 referrals for suspected anemia and 93 for elevated hemoglobin were analyzed. Referral patterns showed significant variance in hemoglobin levels based on different laboratory thresholds. Referrals for suspected anemia in females from laboratories whose lower limit was 120 g/L or greater had a hemoglobin concentration 7.5 g/L greater than referrals that used laboratories with a threshold lower than 120 g/L. The study also identified potential areas for improvement in pre-referral investigations; 44% of eConsults did not provide a ferritin level, 53% were missing a B12 level, and 81% were missing a reticulocyte count. In conclusion, laboratory reference intervals for hemoglobin significantly influence referral patterns for suspected hemoglobin abnormalities in Ontario’s eConsult system. There is a need for standardized reference intervals and comprehensive pre-referral testing to avoid unnecessary medicalization and referrals. We propose an anemia management algorithm to guide primary care providers in the pre-referral investigation process.

## Author summary

Changes in hemoglobin levels, the proteins in red blood cells that carry oxygen around in the body, are common reasons for referrals. In Ontario, Canada, healthcare practitioners have access to electronic consults (eConsults) to ask for specialist advice for various conditions, including suspected anemia (low hemoglobin) or erythrocytosis (high hemoglobin). However, what’s considered ’normal’, ‘high’, and ‘low’ hemoglobin levels can vary from one lab to another. Our research looked at how these differences affect physicians’ decisions to request an eConsult. We found that when labs set higher ’normal’ levels for hemoglobin, doctors were more likely to ask for a specialist opinion. We also noticed that many eConsults did not include all test results needed for an effective eConsult. We believe that having a common standard for hemoglobin intervals and making sure all necessary tests results are included could prevent unnecessary eConsults and lead to higher quality advice from specialists. To help with this, we’ve come up with a guide for doctors to use when deciding to send an eConsult, aiming to make care more efficient and convenient for patients.

## Introduction

Electronic consults (eConsults), a form of digital healthcare delivery, allow physicians and nurse practitioners in Ontario, Canada to access specialist advice, resulting in reduced wait times for patients and improved patient and provider satisfaction [1,2]. Additionally, eConsults offer cost-savings to the healthcare system. One Canadian study analyzed the costs of delivery, specialist fees, and face-to-face referrals prompted by eConsults against the savings from avoided traditional referrals, travel, and lost wages. Out of 3487 eConsults, 40% avoided face-to-face visits, leading to net societal savings of $38,729, or $11 per eConsult [3]. Further, an American study compared cost of eConsults to in-person consultations across 11 specialties. They reported cost savings at the 3-month mark and found that hematology eConsults saved $892 over 3 months, and other savings ranged from $120 in cardiology to $1100 in oncology [4].

In Canada, anemia is the most common cause for hematology consultation via eConsults, but there is no agreed-upon hemoglobin concentration that defines anemia [5,6]. Specifically, despite the ubiquity of “anemia” in primary care practice, the thresholds to define hemoglobin reference intervals remain the subject of considerable debate [7–10]. In the absence of a consensus criteria for anemia, most physicians rely on a given laboratory reference interval.

One of the earliest criteria for anemia, published by the World Health Organization (WHO) in 1958, was limited by small sample size and lack of age, gender, and ethnic diversity [11]. Despite these shortcomings, the WHO’s lower limit of normal, 130 g/L for males and 120 g/L for females, has been widely adopted, raising concerns about applicability across subgroups. For example, studies demonstrate variations in hemoglobin levels among different ethnic groups, with some research suggesting that certain populations, including individuals of Black and Asian descent, may have lower normal hemoglobin levels compared to White individuals [7,8]. Regarding sex differences, some argue that the lower limit should be more uniform for both sexes, attributing observed differences to iron losses from menstruation in females, which ought to be addressed therapeutically [9]. Finally, age-related variations in hemoglobin levels have been investigated, demonstrating a normal decrease in mean hemoglobin in men and women with aging [12–14]. Two widely used private laboratories in Canada differ in their reference intervals and do not adjust hemoglobin levels for older adults, potentially resulting in the medicalization of healthy older adults [9–10].

In this study, we investigated referral patterns of patients with hemoglobin abnormalities referred as eConsults in Ontario, Canada. The primary objective was to determine whether the reference intervals of the reporting laboratory influenced referrals. In so doing, we aimed to assess whether individual laboratory references intervals may contribute to medicalization and unnecessary referrals. The secondary objective was to assess the general investigations performed for hemoglobin abnormalities by primary care prior to referral. Understanding electronic referral patterns is key to providing guidance on the investigation and management of patients with suspected anemia or elevated hemoglobin concentrations in primary care.

## Methods

### Study population and design

Data were collected from Ontario Telemedicine Network’s platform for eConsults assessed by members of the Division of Hematology (BCY, ICY, CCH) at the London Health Sciences Centre (LHSC) between July 1, 2019 to June 30, 2023. Their comments and corresponding laboratory reports were assessed to identify eConsults for low hemoglobin (suspected anemia) or elevated hemoglobin. Excluded were eConsults for patients under 18 years of age, other hematology referrals such as those with multiple cell-line abnormalities, or those pertaining to treatment inquiries. This step allowed us to narrow the clinical context by removing referrals where anemia or erythrocytosis were secondary to the reason for referral, such as referrals about multiple myeloma, chronic myeloid leukemia, and myelodysplastic syndrome. A prespecified subgroup of eConsults made from July 1, 2022, to December 31, 2022 had additional data collected, including prior hemoglobin level, ferritin, mean corpuscular volume (MCV), reticulocyte count, and vitamin B12 level.

### Statistical analyses

The data were sorted into sex-based cohorts and categorized based on hemoglobin reference interval cut-offs for the laboratory-specific lower limit of normal (LLN) and upper limit of normal (ULN). Within referrals for suspected anemia, males were divided into two groups: those with a LLN less than 135 g/L and those with a LLN equal to or greater than 135 g/L. Similarly, females were divided into two groups: those with a LLN less than 120 g/L and those with a LLN equal to or greater than 120 g/L. Among referrals for elevated hemoglobin, males were divided by an ULN threshold of 175 g/L, and females by an ULN threshold of 150 g/L.

Statistical normality was assessed using the Shapiro-Wilk test, and a two-sided Mann-Whitney U-test was used to compare means between groups. All analyses were performed using R version 4.2.1. A p-value threshold of 0.05 was considered statistically significant. This study was approved by the Research Ethics Boards at Western University (ID: 123148).

## Results

A total of 619 consecutive eConsult referrals were reviewed, and after the exclusion criteria were applied, the final study sample included 251 referrals for suspected anemia (127 male; 124 female), and 93 referrals for elevated hemoglobin (59 male, 34 female). Demographic and clinical data of the study population are presented in Table 1. Referrals were made from throughout Ontario, Canada, and variability in reference intervals for males and females was seen across the different laboratories (see Table 2). Referring clinicians included 300 family physicians, 44 nurse practitioners, and 7 specialists.

**Table 1:** Demographic and clinical characteristics of the study population.

Characteristic	Anemia (n = 251)		Elevated hemoglobin (n = 93)	
	Male (n = 127)	Female (n = 124)	Male (n = 59)	Female (n = 34)
Mean age–years ± SD	66 ± 16	54 ± 20	54 ± 16	62 ± 15
Mean Hb–g/L ± SD	123 ± 10.6	108 ± 10.1	178 ± 9.2	162 ± 7.7
LLN–n (%)				
High LLN	103 (81.1)	107 (86.3)	–	–
Low LLN	24 (18.9)	17 (13.7)	–	–
ULN–n (%)				
High ULN	–	–	31 (52.5)	21 (61.8)
Low ULN	–	–	28 (47.5)	13 (38.2)

SD = standard deviation; Hb = hemoglobin; LLN = lower limit of normal; ULN = upper limit of normal; High LLN means ≥ 135 g/L for males and ≥ 120 g/L for females, Low LLN means <135 g/L for Males and <120 g/L for females; High ULN means ≥ 175 g/L for males and ≥ 150 g/L for females; Low ULN means < 175 for males and <150 for females.

**Table 2:** Sex-specific hemoglobin reference intervals from eConsults in Ontario, Canada.

Hb Reference Interval (g/L)	Referrals*
**Females**	
110–147	19
110–150	1
115–155	4
115–160	4
115–165	2
120–153	2
120–160	97
**Males**	
129–165	34
130–170	1
130–180	2
132–170	1
135–165	5
135–170	2
135–175	109
140–175	1

Hb = hemoglobin

*60 eConsults were excluded in this table because either the lower or upper limit of reference interval was not available in the data collection.

### Anemia referrals

**Fig 1:**
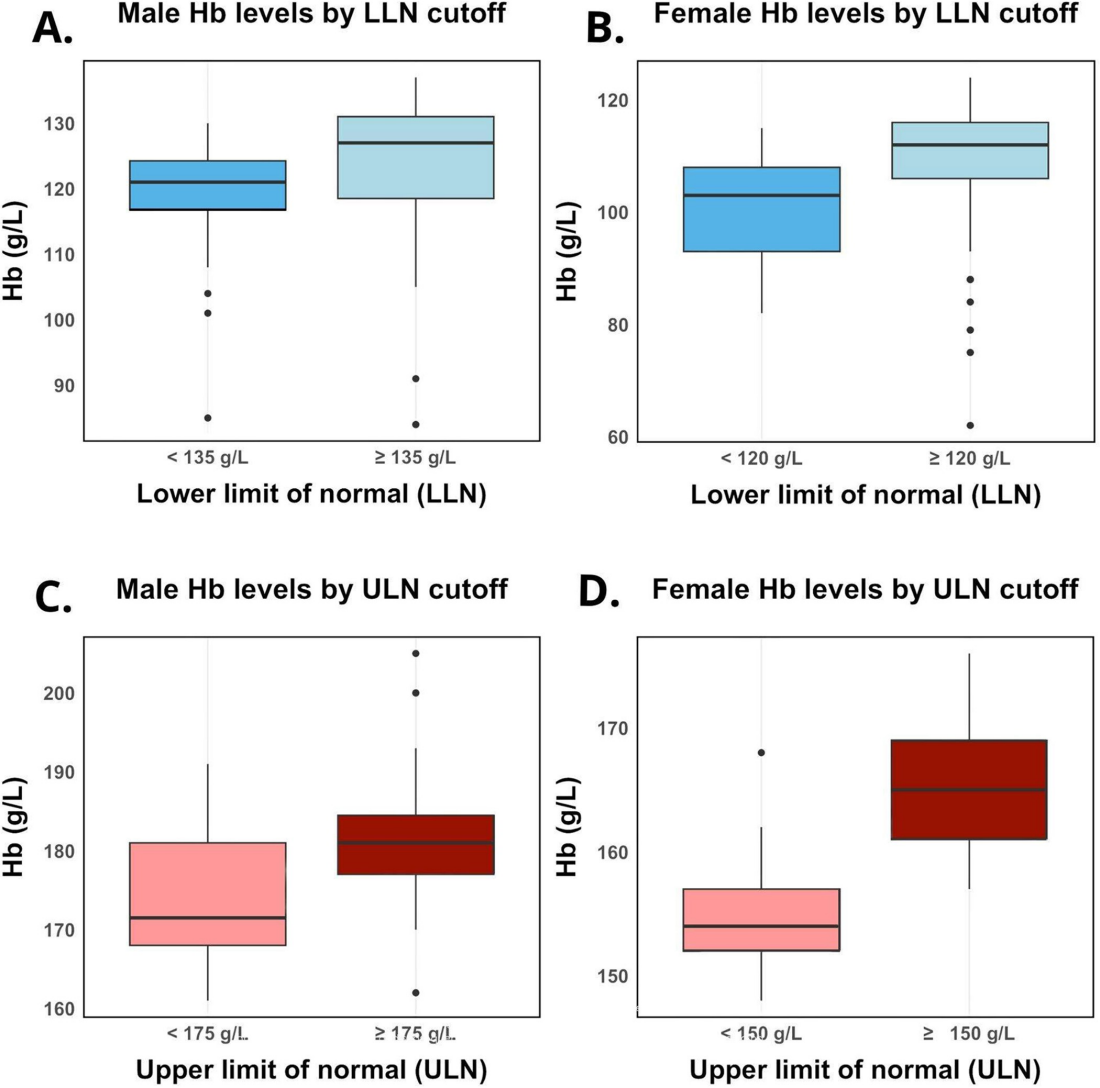
Distribution of hemoglobin levels by sex and normal range thresholds. Panels A and B depict hemoglobin levels for anemia referrals for males and females, respectively, sorted by LLN. Panels C and D depict hemoglobin levels for elevated hemoglobin referrals for males and females, respectively, sorted by ULN threshold. Hb = hemoglobin; LLN = lower limit of normal; ULN = upper limit of normal.

In males referred for suspected anemia, there were 103 with a LLN of 135 g/L or greater, and 24 with a LLN of less than 135 g/L. The mean hemoglobin level for the group with a LLN of ≥135 g/L was 123.7 g/L, significantly higher (p = 0.003) than the 118.4 g/L observed in the group with a LLN of <135 g/L (Fig 1A). In females referred for suspected anemia, there were 107 with a LLN of 120 g/L or greater, and 17 with a LLN of less than 120 g/L. The mean hemoglobin level for the group with a LLN of ≥120 g/L was 109.1 g/L, significantly higher (p<0.001) than the 101.6 g/L observed in the group with a LLN of <120 g/L (Fig 1B).

### Elevated hemoglobin referrals

Among males referred for elevated hemoglobin, there were 31 with an ULN of 175 g/L or greater, and 28 with an ULN of less than 175 g/L. The mean hemoglobin level for the group with an ULN of ≥175 g/L was 182.0 g/L, significantly higher (p = 0.002) than the 174.3 g/L observed in the group with an ULN of <175 g/L (Fig 1C). Among females referred for elevated hemoglobin, there were 21 with an ULN of 150 g/L or greater, and 13 with an ULN of less than 150 g/L. The mean hemoglobin level for the group with an ULN of ≥150 g/L was 165.5 g/L, significantly higher (p<0.0001) than the 155.2 g/L observed in the group with an ULN of <150 g/L (Fig 1D).

### Additional hematological parameters

There were 81 eConsults for anemia between July 1, 2022, to December 31, 2022 which had additional data collected including demographic and clinical data (see Table 3). There were 18 microcytic, 56 normocytic, and 5 macrocytic anemia referrals with 2 referrals that did not report the MCV. Additionally, 44% did not provide a ferritin level, 53% were missing a B12 level, and 81% were missing a reticulocyte count. In a post-hoc analysis, we used public physician and nurse registries to determine the number of years of practice of each referring clinician based on when they were granted their independent practice license. There was no significant difference in the number of years of practice between referrals where ferritin, B12, or reticulocyte count were provided or not. Regarding prior test results, 20% did not provide a prior hemoglobin level; among those that did, the mean change between the prior and most recent hemoglobin was a decrease by 1.8 g/L.

**Table 3:** Demographic and clinical characteristics of the subset population with additional hematological data collected.

Characteristic	Anemia (n = 81)	
	Male (n = 43)	Female (n = 38)
**Mean age–years ± SD**	66 ± 17	52 ± 19
**Mean Hb–g/L ± SD**	123 ± 11	108 ± 10
**MCV–n (%)**		
MCV < 80 fL	2 (5)	16 (42)
MCV 80–100 fL	35 (82)	21 (55)
MCV > 100 fL	5 (12)	0 (0%)

Hb = hemoglobin; MCV = mean corpuscular volume; missing data included 66 reticulocyte counts (81%), 41 vitamin B12 (54%), 36 ferritin (44%), and 16 prior hemoglobin (20%), and 2 prior MCV values (2%).

## Discussion

Our study of practice referral patterns to provincial virtual care in hematology identified several important findings. Our null hypothesis was that hemoglobin concentration would not vary between referrals based on the LLN or ULN used by particular laboratories. Our results rejected this, demonstrating significant differences in hemoglobin concentrations between referrals made from laboratories with different LLN or ULN. For example, females referred for suspected anemia from laboratories whose LLN was 120 g/L or greater had a hemoglobin concentration 7.5 g/L higher than those referred from laboratories with a threshold lower than 120 g/L. Assuming the populations of patients attending laboratories are similar, the variation in hemoglobin concentration across reference intervals indicate that laboratory-specific cut-offs may be influencing the decision to refer. This is particularly significant given that up to 64% of clinical decisions are guided by laboratory testing, underscoring the profound impact of these laboratory thresholds on clinical practice [15]. Ideally, the decision to refer, and the suspicion of disease, in general, should be grounded in evidence and not be swayed by arbitrary thresholds that may vary between laboratories and are not consistently underpinned by rigorous evidence. The results suggest that there might be an over-reliance on these laboratory-set cut-offs, potentially leading to a skew in referrals based on laboratory-defined norms rather than clinical evidence and patient symptoms.

### Physiologic variation in hemoglobin

A variety of physiological and lifestyle factors can influence hemoglobin levels, usually transiently, resulting in abnormal but non-pathological variations. A study of male athletes found that exercise increased hemoglobin concentration by 4.6 g/L, and this effect subsided approximately two hours after the exercise was completed [16]. Research suggests this effect is mediated by the well-studied haemoconcentration effect of dehydration [17]. Conversely, there are non-pathological factors that may mildly decrease hemoglobin levels and trigger otherwise unnecessary investigations or referrals for anemia. Studies have found a diurnal pattern in hemoglobin levels where hemoglobin gradually decreases during the course of the day, with the lowest levels occurring around midnight [18,19]. Additionally, post-prandial reductions in red blood cell count, hemoglobin concentration, and hematocrit level have been reported, with a decrease in hemoglobin concentration by 8% [20]. Given these multiple physiological influences on hemoglobin, the take-home message is not necessarily to work up and rule out each of these, but rather to emphasize the importance of a thorough history, physical exam, and ordering repeat bloodwork.

### Investigating hemoglobin abnormalities

Further, while immediate work up and referral are appealing to both clinicians and patients to provide reassurance, there is often a window for observation; a study by Slusar et al. in 2017 revealed that when managed with a letter to the referring physician, 80% of referrals to hematology concerning mild cytopenias and gammopathies, as well as elevated ferritin levels, had either self-resolved or were stable at 1-year follow-up [21]. Having a prior hemoglobin value and understanding the pattern of chronicity may help with identifying an underlying cause of hemoglobin abnormalities and its urgency.

**Fig 2:**
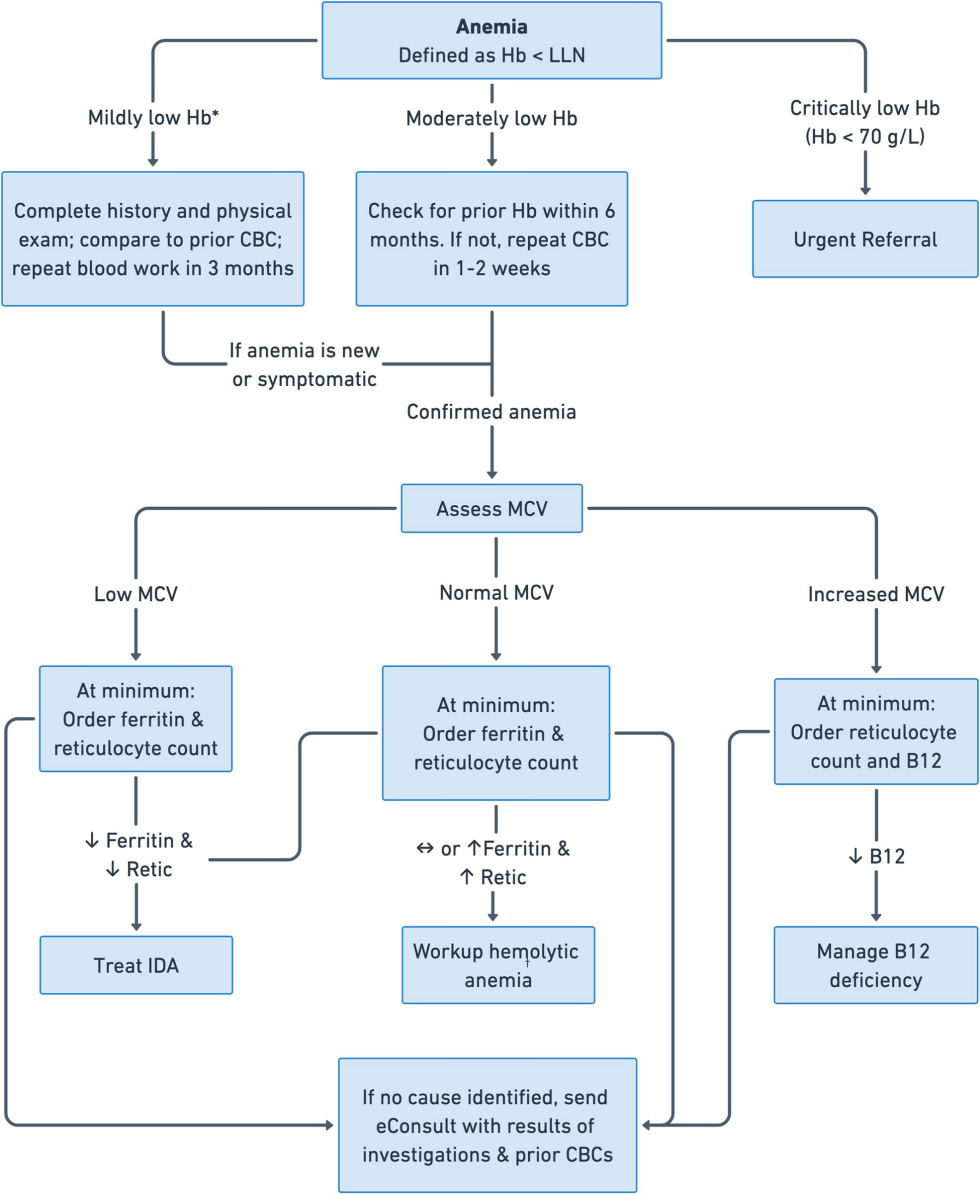
Algorithm for the management of anemia prior to consultation. Hb = hemoglobin; LLN = lower limit of normal; CBC = complete blood count; MCV = mean corpuscular volume; IDA = iron deficiency anemia. *There is no uniform definition of mild anemia. We define mild anemia here as hemoglobin 100–119 g/L for females and 100–129 g/L for males similar to Tettamanti et al [22]. ^†^Workup for hemolytic anemia should include serum lactate dehydrogenase (LDH), indirect bilirubin, haptoglobin and a peripheral blood film.

Analysis of referral patterns for anemia and work up prior to eConsults in our study showed varied and inconsistent pre-referral investigations, suggesting an area for potential improvement in the quality of referrals. Notably, important measures such as iron studies (ferritin, transferrin saturation), vitamin B12 levels and reticulocyte count are often useful in the investigation of patients with anemia prior to referral. We acknowledge the constraints faced by primary care physicians in terms of time and limited follow up opportunities that make balancing the number of tests and timing of referrals challenging. Given in-person consultations by Hematologists have prolonged wait times, often several months depending on the centre, the virtual care opportunity via eConsults provides faster access to specialist opinions. In either case, providing more information to the consulting service, especially through a digital platform, will allow a more meaningful discussion and potentially even annul the need for referral in the first place. Cross-sectional analyses of various regions in our province have found that 46% - 51% of eConsults result in a referral being avoided that was originally contemplated [1,5]. To this end, we suggest an algorithm for the management of anemia prior to consultation (see Fig 2).

### Limitations

This study has several limitations. Being retrospective and cross-sectional in nature, this study cannot capture the progression of clinical scenarios, management decisions made over time, nor the outcomes of those decisions. Additionally, the data reflect only eConsults to the Division of Hematology in London, Ontario, which reflect much of Southwestern Ontario, but may not be representative of broader provincial or national practice patterns, nor reflect patterns of traditional in-person referrals. Although our study identified gaps in pre-referral testing, we did not investigate why these gaps exist, and so future research into the reasons behind these gaps would be informative. Further research could benefit from a prospective design, broader geographic sampling, tracking patient outcomes, and inclusion of referring physician satisfaction to enhance the depth and applicability of the findings. Additionally, future studies investigating the cost-effectiveness or potential cost-savings of implementing standardized hemoglobin reference intervals could provide valuable insights into the economic impact of such standardization.

## Conclusion

Our investigation suggests that laboratory set thresholds may influence referrals of hemoglobin abnormalities and that virtual care referrals can be further enhanced with additional pre-referral laboratory testing. There are several potential solutions to address these issues. Beginning upstream, hemoglobin thresholds should be revisited and redefined to accurately represent diverse patient populations, ensuring that non-pathological variances are not flagged as abnormal. Concurrently, when abnormalities are found, it is important to ensure preliminary investigations, as well as monitoring when appropriate, are conducted before patients are referred to specialists. To assist with preliminary investigation and monitoring, we suggest an algorithm to guide anemia management in the primary care setting.
